# Impact of SNPs in *ACACA*, *SCD1,* and *DGAT1* Genes on Fatty Acid Profile in Bovine Milk with Regard to Lactation Phases

**DOI:** 10.3390/ani10060997

**Published:** 2020-06-08

**Authors:** Marzena M. Kęsek-Woźniak, Edyta Wojtas, Anna E. Zielak-Steciwko

**Affiliations:** Institute of Animal Breeding, Wroclaw University of Environmental and Life Sciences, 50-375 Wroclaw, Poland; marzena.kesek@gmail.com (M.M.K.-W.); edyta.wojtas@upwr.edu.pl (E.W.)

**Keywords:** fatty acid, milk, *ACACA*, *SCD1*, *DGAT1*, SNPs, cattle

## Abstract

**Simple Summary:**

Fatty acids are an important component of milk fat. Because of their wide spectrum of effects on human health, it is important to better understand the regulation of their profile in milk. This study aims to analyzing the relation between selected genes with milk fatty acid content. As increased concentration of unhealthy fatty acids and lower concentration of healthy ones in less frequent homozygotes and a strong influence of the genes on fatty acids with 18 carbon atoms were observed, these findings could be useful in future dairy cattle selection aiming production of more healthy milk.

**Abstract:**

Milk fat is a dietary source of fatty acids (FA), which can be health promoting or can increase risks of some diseases. FA profile composition depends on many factors, among them gene polymorphism. This study analyzed the relation between polymorphism of acetyl-CoA carboxylase α (*ACACA*), stearoyl-CoA desaturase 1 (*SCD1*), diacylglycerol acyltransferase 1 (*DGAT1*) genes with FA profile in milk from Polish Holstein-Friesian cattle and determined changes of FA percentage during lactation with regard to polymorphism. Milk samples were collected twice: during the first phase of lactation (<90 Days in milk; DIM) and at the end of lactation (>210 DIM). During the first milk collection, blood samples were taken to analyze three chosen single nucleotide polymorphisms (SNPs): AJ312201.1g.1488C > G SNP in *ACACA* gene, A293V SNP in *SCD1* gene, and K232A SNP in *DGAT1* gene. Increased concentration of FA that are less beneficial for human health and have lower concentration of healthy FA in homozygotes: GG in *ACACA*, VV in *SCD1*, and KK in *DGAT1* were observed, as well as a strong influence of the analyzed genes on FA with 18C atoms was also found. Moreover, it was demonstrated that lactation phase significantly affected FA percentage in milk depending on the phenotype. These results may contribute their part to knowledge toward obtaining more beneficial milk composition.

## 1. Introduction

Bovine milk is an important component of human diet. Its influence on health depends on several factors, including fat composition, as some fatty acids (FA) are known to be health promoting and other increasing risk of some diseases [1]. In an updated review, Gómez-Cortés et al. [2] suggested that milk fat consumed as whole dairy product can support human health owing to some fatty acids connected to dairy matrix.

There are two sources of fatty acids in milk: *de novo* synthesis in milk-producing cells and transfer from blood via the milk-producing cells. *De novo* synthesis of FA is a multistep process catalyzed by several enzymes that are encoded by different genes [3]. In cattle, two genes are located on chromosome 19: acetyl-CoA carboxylase α (*ACACA*) gene encoding acetyl-CoA carboxylase α [4] and fatty acid synthase (*FASN*) gene encoding fatty acid synthase [5]. On chromosome 26, stearoyl-CoA desaturase 1 (*SCD1*) gene is located and it encodes stearoyl-CoA desaturase [6], while on chromosome 14 diacylglycerol acyltransferase 1 (*DGAT1*) gene, encoding diglyceride acyltransferase 1, is located [7]. It was described that the FA synthesized *de novo* have moderate to high heritability estimates, while those transferred from blood are characterized by low to moderate heritability estimates as their content depends also on non-genetic factors such as feeding [8]. Moreover, fatty acids present high variability mostly because of the *DGAT1* gene [9].

It is known that some gene polymorphism in cattle affect FA profile in milk. A nonsynonymous mutation (A293V) in *SCD1* gene involving a substitution of T to C and changing valine to alanine was described by several authors. Duchemin et al. [10] demonstrated that allele V was negatively associated with some health promoting FA. Bouwman et al. [11] explain that this SNP influence on medium-chain unsaturated fatty acids (UFA) and their saturated fatty acids (SFA) equivalents is coherent with the activity of the encoded enzyme. Another well-known polymorphism affecting FA profile in milk is a nonsynonymous non-conservative substitution of lysine to alanine, the K232A SNP in *DGAT1* gene. A significantly lower content of some health-oriented C18 FA with presence of allele K was described by Bovenhuis et al. [12]. Limited number of research was conducted in bovine *ACACA* gene. Even though, Li et al. [13] demonstrated a QTL significantly associated with some FA with this gene. Also, Pegolo et al. [14] confirmed that polymorphism in *ACACA* is related to some *de novo* synthetized fatty acids. This type of research contribute to knowledge on how combination of different phenotypes affect milk fatty acid composition which could be used in the future as a component of genetically controlled milk production aiming health-promoting milk.

The purpose of the presented research is analyzing the influence of *ACACA*, *SCD1*, *DGAT1* genes polymorphism on the fatty acid profile in milk from Polish Holstein-Friesian cattle and determining the changes of fatty acids content during lactation depending on polymorphism of the analyzed genes.

## 2. Materials and Methods

All experimental procedures were licensed by the 2nd Local Ethics Committee at Wrocław University of Environmental and Life Sciences, Poland.

### 2.1. Animals and Management

The material consisted of individual blood (*n* = 144) and milk samples (*n* = 288) from Polish Holstein-Friesian cows in lactations from 1st up to 5th. The first milk sampling was done in the first phase of lactation (<90 Days in milk; DIM) during the evening milking (*n* = 144). Milk samples were taken for a second time in the last phase of lactation (>210 DIM), also during the evening milking (*n* = 144). Blood samples were collected only once, in the course of the first milk sampling, in EDTA containers. The entire herd of Polish-Holstein dairy cattle was kept in the same farm located in the South-West region of Poland. Animals had somatic cell count (SCC) < 400,000 cells/mL. Cows were fed with total mixed ration with corn silage as primary component (no pasture).

### 2.2. Experimental Design

The variables consisted of fat, protein, and lactose content, individual FA from C4:0 to C20:4 ω-6, and following classes of FA: SFA, MUFA, PUFA, UFA, SCFA (short-chain fatty acids; ≤6 carbon atoms in chain), MCFA (medium-chain fatty acids; 8–17 carbon atoms in chain), LCFA (long-chain fatty acids; ≥18 carbon atoms in chain), and trans FA (all detected trans isomers). C11:0 and C15:1 were excluded from the analysis because of a very low concentration in the analyzed samples.

The index of desaturation (DI) was calculated for the individual fatty acids with the following formulas (adapted from Conte et al. [14]): DI C14 = C14:1 cis-9 / (C14:1 cis-9 + C14:0); DI C16 = C16:1 cis-9 / (C16:1 cis-9 + C16:0); DI C18 = C18:1 cis-9 / (C18:1 cis-9 + C18:0).

Protein, fat, lactose concentration were analyzed in all milk samples using Infrared Milk Analyzer 150 (Bentley Instruments Inc., Chaska, MN, USA), while SCC using Somacount 150 apparatus (Bentley Instruments Inc.). Milk fat was extracted according to Folch method [15]. Esterification was completed with the use of potassium hydroxide in methanol-hexane. The fatty acid composition was examined using 7890 gas chromatograph with flame ionization detector (Agilent Technologies, Santa Clara, CA, USA) as described by Kęsek et al. [16].

In the presented study, the polymorphism in *ACACA* gene is indicated with the nucleotide name, while the single nucleotide polymorphism (SNP) in *SCD1* and *DGAT1* genes are indicated using the name of the coded amino-acid. DNA was isolated from blood with GenElute™ Blood Genomic DNA Kit (Sigma Aldrich, St. Louis, MO, USA) according to producer’s protocol. Concentration and purity of all DNA samples were evaluated using NanoDrop 2000c spectrophotometer (Thermo Fisher Scientific, Wilmington, DE, USA). For polymorphism analysis, the AJ312201.1g.1488C > G (g.1488C > G) SNP in *ACACA* gene [16] and the well-know A293V SNP in *SCD1* gene were chosen. The samples were analyzed by PCR-RFLP as described by Kęsek et al. [16].

The K232A SNP in *DGAT1* gene was chosen as the third SNP for analysis. The detection of this SNP was conducted according to Steinberg et al. [17] with modifications. The method was tetra-primer amplification refractory mutation system-PCR (ARMS-PCR). The system is based on four primers:

Internal primers: Ala-Rev—5′-agctcccccgttggccgc-3′; Lys-For—5′-tcgtagctttggcaggtaagaa-3′,

External primers: Ala-For—5′-gtcaacctctggtgccgagag-3′; Lys-Rev——5′-cacctggagctgggtgaggaa-3′.

The amplification was conducted with use of Maxima™ Hot Start Green PCR Master Mix (2X) (Thermo Fisher Scientific). In each reaction, the individual DNA sample used for amplification contained 25 to 100 ng of genomic DNA. Before amplification, four primer mixtures were prepared: 0.2 µM mixture of DGAT1-Ala-For, 0.2 µM mixture of DGAT1-Lys-Rev, 0.8 µM mixture of DGAT1-Ala-Rev, and 0.8 µM mixture of DGAT1-Lys-For. The PCR mixture of 25 µL was prepared: Master Mix 12.5 µL, 1 µL of each primer mixture, 3 µL of DNA, 5.5 µL water. The procedure was conducted as follows: initial denaturation (94 °C for 5 min), 35 PCR cycles (94 °C for 30 s; 65 °C for 30 s; 72 °C for 30 s), final elongation (72 °C for 5 min). The amplification products were separated by electrophoresis on 1.5% ethidium bromide-stained agarose gel for 30 min at 100 V.

### 2.3. Statistical Analysis

To perform the statistical analysis the animals were grouped as follow: *ACACA* polymorphism —CC, CG, GG; *SCD1* polymorphism—AA, VA, VV; *DGAT1* polymorphism—AA, KA, KK; lactation phase—I (<90 DIM), II (>210 DIM). The χ2 test was used to verify polymorphisms and alleles frequencies with Hardy-Weinberg’s equilibrium. The polymorphism is represented by the number and frequency of occurrence. The remaining continuous variables, such as fatty acid profile and milk composition, are presented using the distribution parameters: mean, standard deviation (SD) in the text or standard error (SE) in Figures. Differences in the analyzed parameters, for each lactation phase separately, depending on polymorphism were tested by the ANOVA Kruskal-Wallis test with a post hoc test of multiple comparisons. Differences in the analyzed parameters between lactation phases within polymorphism were analyzed using the Wilcoxon Signed Rank Test. Calculations and analyses were conducted using the STATISTICA 13 package (StatSoft Polska Sp. z o.o., 30-110 Kraków, Poland). Differences between group means were tested for significance (*p* < 0.05; *p* < 0.01).

## 3. Results

Most of animals were homozygous: CC in *ACACA* gene, AA in *SCD1* gene, and AA in *DGAT1* gene (Table 1). No significant deviation from Hardy-Weinberg’s equilibrium was found for all SNP.

### 3.1. Polymorphism Effect on Fatty Acid Profile

#### 3.1.1. *ACACA* Gene Effect

**Figure 1:**
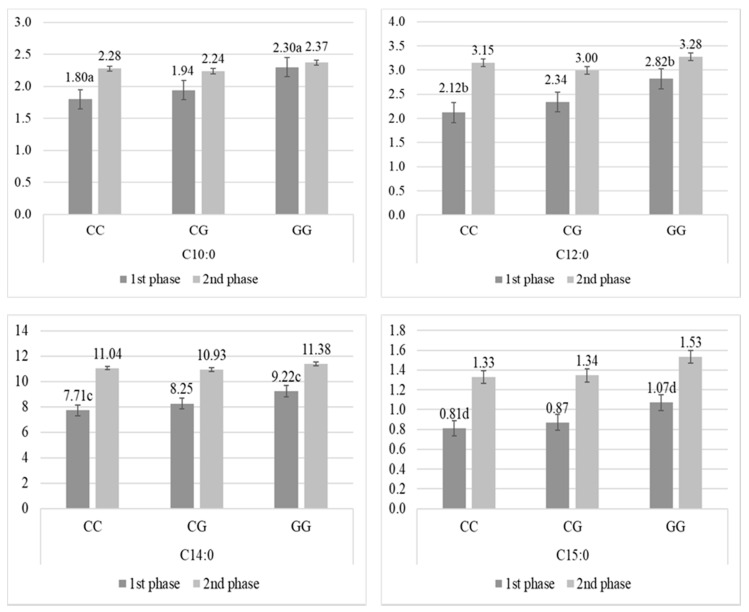
Percentage (mean ± SE) of C10:0, C12:0, C14:0, and C15:0 fatty acids in milk from Polish Holstein-Frisian cows during first and second phase of lactation depending on AJ312201.1g.1488C > G (g.1488C > G) single nucleotide polymorphism (SNP) in acetyl-CoA carboxylase α (*ACACA*) gene. a, b, c, d—values differ significantly between polymorphisms within fatty acid (*p* < 0.05).

Statistically significant differences for g.1488C > G SNP were found only between the homozygotes for several fatty acids and their traits. Interestingly, these significant differences in FA amount in milk fat were observed only in the first phase of lactation. The amount of C10:0, C12:0, C14:0, and C15:0 were higher with presence of G allele, cows with GG had the highest content of these acids compared to cows with GC and CC (Figure 1), but significant differences were observed only between homozygotes (*p* < 0.05). Moreover, cows with GG had the highest content of MCFA (50.52% ± 4.72) compared to cows with GC (47.38% ± 4.36) and CC (46.01% ± 4.22), but significant differences were observed only between homozygotes (*p* < 0.05).

**Figure 2:**
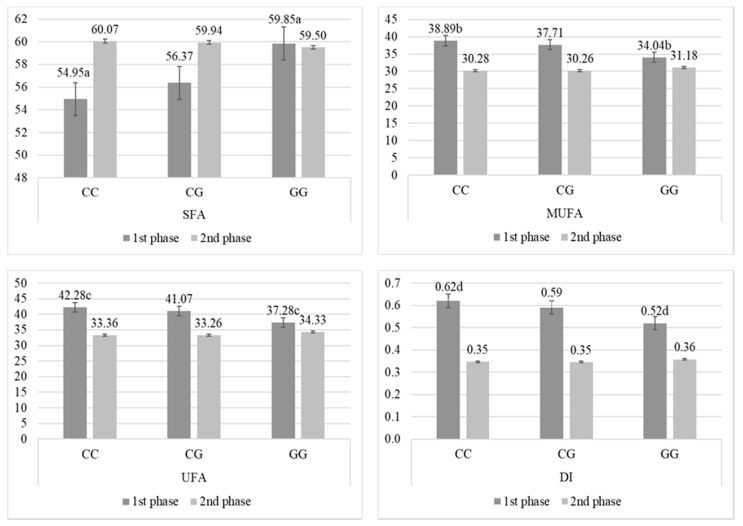
Percentage (mean ± SE) of saturated fatty acids (SFA), monounsaturated fatty acids (MUFA), unsaturated fatty acids (UFA), and index of desaturation (DI) in milk from Polish Holstein-Frisian cows during first and second phase of lactation depending on AJ312201.1g.1488C > G (g.1488C > G) single nucleotide polymorphism (SNP) in acetyl-CoA carboxylase α (*ACACA*) gene. a, b, c, d—values differ significantly between polymorphisms within fatty acid (*p* < 0.05).

Analysis of FA unsaturation showed that in the first phase of lactation, cows with GG had also the highest concentration of SFA and the lowest content of MUFA and UFA, as well as DI, compared to cows with CG and GG (Figure 2), but significant differences were observed only between homozygotes (*p* < 0.05).

#### 3.1.2. *SCD1* Gene Effect

**Figure 3:**
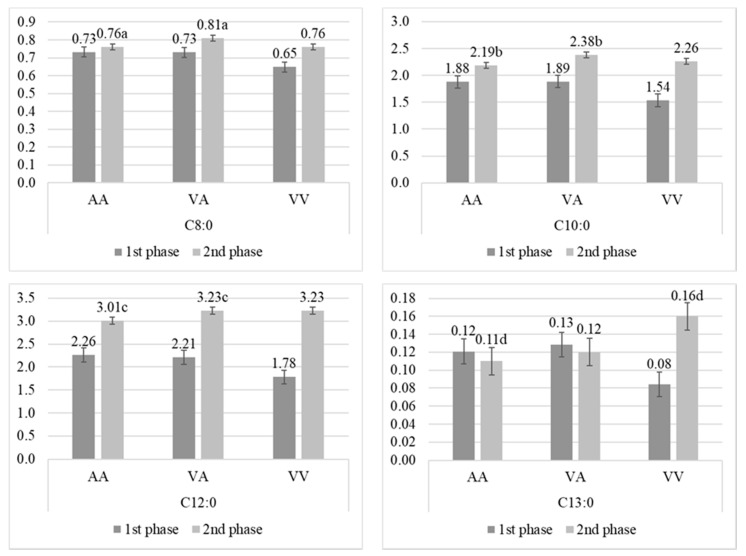
Percentage (mean ± SE) of C8:0, C10:0, C12:0, and C13:0 fatty acids in milk from Polish Holstein-Frisian cows during first and second phase of lactation depending on A293V single nucleotide polymorphism (SNP) in stearoyl-CoA desaturase (*SCD1*) gene. a, b, c, d—values differ significantly between polymorphisms within fatty acid (*p* < 0.05).

Statistically significant differences for A293V SNP were found for several fatty acids during the second phase of lactation (Figure 3). The amount of C8:0 and C10:0 differed depending on polymorphism, cows with VA had the highest content of these acids compared to homozygotes, but significant differences were observed only between AA and VA (*p* < 0.05). Also, cows with AA had the lowest content of C12:0 and C13:0 in this phase of lactation compared to VA and VV, but significant differences (*p* < 0.05) were observed only between AA and VA for C12:0, and AA and VV for C13:0.

**Figure 4:**
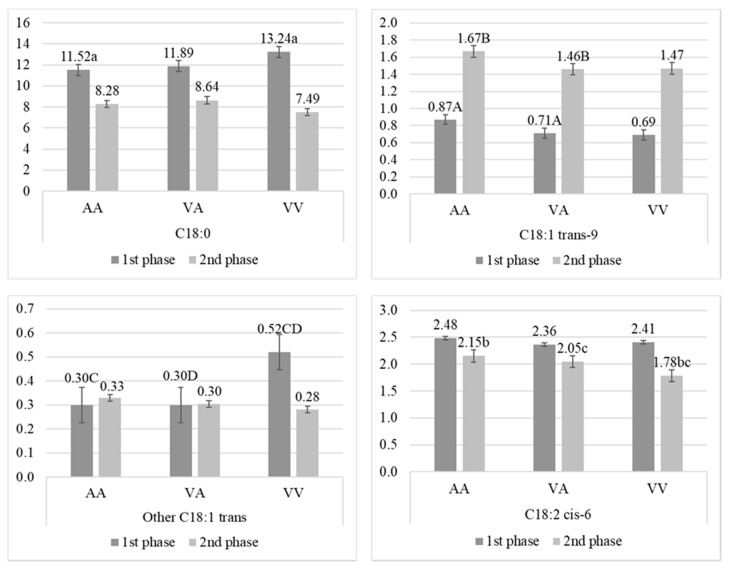
Percentage (mean ± SE) of fatty acids with 18 carbon atoms in milk from Polish Holstein-Frisian cows during first and second phase of lactation depending on A293V single nucleotide polymorphism (SNP) in stearoyl-CoA desaturase (*SCD1*) gene. a, b, c, d—values differ significantly between polymorphisms within fatty acid (*p* < 0.05); A, B, C, D—values differ highly significantly between polymorphisms within fatty acid (*p* < 0.01).

Moreover, the group of fatty acids with chain of 18 carbon atoms was found to be related to A293V SNP (Figure 4). The saturated form, C18:0, was found in abundance in milk from cows with VV during the first phase of lactation, but significant differences (*p* < 0.05) were observed only between VV and AA. In both phases, cows with AA had higher concentration of C18:1 trans-9 in milk fat than cows with VA and VV (which were similar), but highly significant differences (*p* < 0.01) were observed only between AA and VA. In opposite, the highest concentration (*p* < 0.01) of other C18:1 trans FA was detected in milk from cows with VV during the first phase of lactation, while in the second phase this concentration aligned with AA and VA. Also, cows with VV had significantly (*p* < 0.05) lower concentration of C18:2 cis-6 during the second phase of lactation.

Only the content of PUFA during the second phase of lactation was found to be related to the SNP (AA—3.12% ± 0.52; VA—2.99% ± 0.42; VV—2.63% ± 0.18), but significant differences (*p* < 0.05) were observed only between AA and VA. Also, DI C14 was found to be linked to this SNP. Cows with AA had higher DI C14 in both lactation phases (1st phase: AA—0.10 ± 0.02; VA—0.08 ± 0.02; VV—0.09 ± 0.02; 2nd phase: AA—0.13 ± 0.02; VA—0.12 ± 0.02; VV—0.12 ± 0.02), but highly significant differences (*p* < 0.01) were observed only between AA and VA.

#### 3.1.3. *DGAT1* Gene Effect

**Figure 5:**
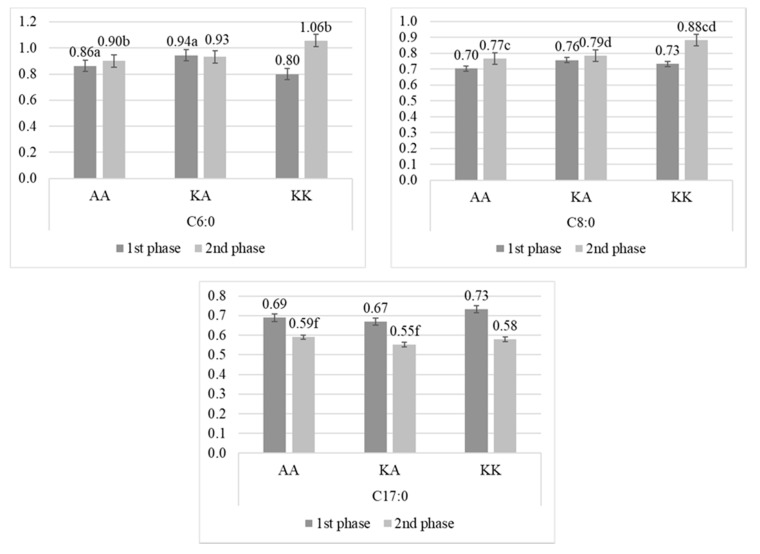
Percentage (mean ± SE) of C6:0, C8:0, and C17:0 fatty acids in milk from Polish Holstein-Frisian cows during first and second phase of lactation depending on K232A single nucleotide polymorphism (SNP) in diglyceride acyltransferase (*DGAT1*) gene. a, b, c, d—values differ significantly between polymorphisms within fatty acid (*p* < 0.05).

The K232A SNP was the only analyzed gene affecting fat and protein content in milk. During the second phase of lactation, cows with AA had less (*p* < 0.01) fat than cows with KA (respectively: 3.77% ± 0.62; 4.14% ± 0.63). Also, cows with AA had lower (*p* < 0.01) protein concentration than cows with KK (respectively: 3.67% ± 0.30; 3.96% ± 0.39). Statistically significant differences related to K232A SNP were found for several fatty acids, mostly during the second phase of lactation (Figure 5). Merely, the amount of C6:0 was lower (*p* < 0.05) during the first phase of lactation in milk from cows with AA compared to cows with KA. During the second phase, cows with KK had the highest (*p* < 0.05) content of C6:0 compared to cows with AA. Also, cows with KK had the highest (*p* < 0.05) content of C8:0 in this phase of lactation compared to KA and AA. The heterozygotes had the lowest content of C17:0 during the second phase, but significant differences (*p* < 0.05) were found only between AA and KA.

The C14:0 and C14:1 fatty acids were found to be related to the SNP. Cows with AA (11.2% ± 0.99) had higher concentration of C14:0 during the second phase compared to cows with KA (10.86% ± 0.91) and KK (10.6% ± 0.64), but significant differences (*p* < 0.05) were observed only between AA and KK. Concerning the C14:1, its highest concentration was found in milk from cows with KA (0.87% ± 0.26) compared to cows with AA (0.76% ± 0.27) and KK (0.73% ± 0.19), but significant differences (*p* < 0.05) were observed only between AA and KA. In addition, the C16:0 and C16:1 content were affected by the SNP, but only during the first phase of lactation. Cows with AA had the lowest content of the saturated form (27.22% ± 1.84), compared to cows with KA (28.29% ± 2.27) and KK (28.15% ± 2.54), but significant differences (*p* < 0.05) were detected only between AA and KA. The highest concentration of the unsaturated form was found in milk from cows with KK (5.26% ± 1.68); cows with KA had its lowest content (3.99% ± 1.28) and cows with AA in between (4.59% ± 1.44); statistically significant differences (*p* < 0.05) were observed between AA and KA, as well as between KA and KK.

**Figure 6:**
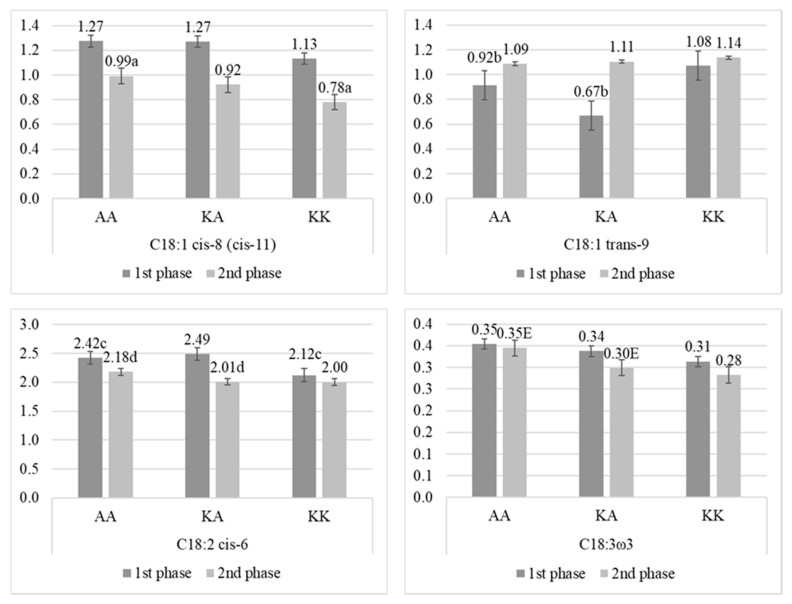
Percentage (mean ± SE) of fatty acids with 18 carbon atoms in milk from Polish Holstein-Frisian cows during the first and second phase of lactation depending on K232A single nucleotide polymorphism (SNP) in diglyceride acyltransferase (*DGAT1*) gene. a, b, c, d—values differ significantly between polymorphisms within fatty acid (*p* < 0.05); A, B, C, D—values differ highly significantly between polymorphisms within fatty acid (*p* < 0.01).

Interestingly, this SNP, likewise the A293V SNP in *SCD1* gene, affected the group of fatty acids with the chain of 18 carbon atoms, but exclusively the unsaturated forms (Figure 6). The lowest concentration of C18:1 cis-8 was found in milk from cows with KK, but significant differences (*p* < 0.05) were found only between AA and KK during the second phase of lactation. The same group of cows (with KK) had also the lowest concentration of C18:2 cis-6 and C18:3ω3. For the first FA, significant differences (*p* < 0.05) were observed between AA and KK during the first phase, and between AA and KA during the second phase. For the second FA, highly significant differences (*p* < 0.01) were observed between AA and KA during the second phase. In opposite, cows with KK had the highest concentration of C18:1 trans-9 in milk fat compared to cows with KA and AA, but significant differences (*p* < 0.05) were observed only between AA and KA in the first phase of lactation.

The DI C16 during the first phase was lower for cows with KA (0.12% ± 0.02) compared to cows with AA (0.14% ± 0.04) and KK (0.16% ± 0.05), but significant differences (*p* < 0.05) were observed only between AA and KA. During both phases, the PUFA content was related to the SNP. In the first phase, the lowest concentration of this group was found in milk from cows with KK (2.97% ± 0.33) compared to cows with AA (3.37% ± 0.42) and KA (3.43% ± 0.39), but highly significant differences (*p* < 0.01) were observed only between AA and KK. Merely in the second phase, cows with KK had its lowest concentration (2.93% ± 0.42) compared to cows with AA (3.16% ± 0.49) and KA (2.94% ± 0.47), but significant differences (*p* < 0.05) were observed only between AA and KA.

### 3.2. Changes of FA Percentage during Lactation Depending on Polymorphism

Lactation phase significantly affected FA percentage and milk composition. Only C13:0, C20:0, C21:1, and DI C18 remained unaffected by all phenotypes during lactation. Detailed results for individual FA and their DI are presented in Supplementary Tables: S1, S2, S3 for each gene separately. SFA concentration increased during lactation, its highest significant increase was observed for CC in *ACACA* (+5.12; *p* < 0.01); VA in *SCD1* (+4.78; *p* < 0.01); and AA in *DGAT1* (+4.94; *p* < 0.01). Contrary to the saturated forms, the UFA concentration decreased during lactation, its highest reduction was observed for CC in *ACACA* (−8.92; *p* < 0.01); VV in *SCD1* (−10.77; *p* < 0.05); and AA in *DGAT1* (−8.5; *p* < 0.01). The same observation was made for MUFA percentage, with highest decrease for CC in *ACACA* (−8.61; *p* < 0.01); VV in *SCD1* (−10.04; *p* < 0.01); and AA in *DGAT1* (−8.29; *p* < 0.01). The highest decrease of PUFA was observed in presence of VV in *SCD1* (−0.73; *p* < 0.01) and KA in *DGAT1* (−0.49; *p* < 0.01), while in case of other phenotypes the decrease was less strong. The changes in SCFA group were affected only by *DGAT1*: a strong increase was observed in presence of KK (+0.55; *p* < 0.05). The MCFA group concentration increased during lactation with phenotype influence, the highest raise was observed for CC in *ACACA* (+10.55; *p* < 0.01), VV in *SCD1* (+15.1; *p* < 0.01), and AA in *DGAT1* (+10.05; *p* < 0.01). The LCFA group was reduced in the second phase of lactation, with the strongest loss observed for VV in *SCD1* (−18.61; *p* < 0.01), while for other phenotypes this decrease was visibly lower and quite similar. The overall DI also declined at the end of lactation with influence of all phenotypes, with the strongest decrease in case of VV in *SCD1* (−0.3; *p* < 0.01). Fat, as could be expected, decreased during lactation. However, in case of CC in *ACACA* (−0.21; *p* < 0.05) and VA in *SCD1* (−0.27; *p* < 0.05) its decrease was the highest comparing to other polymorphisms within genes. Also, AA in *DGAT1* was significantly associated with the reduction of fat in the second phase of lactation (−0.26; *p* < 0.05). Protein concentration were more affected by the analyzed genes during lactation—changes were significant for all phenotypes, but the highest increase of proteins was observed for KK in *DGAT1* (+0.77; *p* < 0.01). In case of lactose, it should be noted that its concentration was not affected by homozygotes with lowest frequency: GG in *ACACA*, VV in *SCD1*, and KK in *DGAT1*. In presence of other phenotypes, the highest decrease of lactose in milk during lactation was observed for VA in *SCD1* (−0.22; *p* < 0.01).

## 4. Discussion

### 4.1. Polymorphism Effect on Fatty Acid Profile

#### 4.1.1. *ACACA* Gene Effect

There are not many studies concerning *ACACA* gene effect on milk traits in cattle. Matsumoto et al. [4] demonstrated that 4 SNP in *ACACA* in Holstein-Frisian cattle showed significant impact on FA profile in milk, mostly on C14:0, C16:0, and C18:0. Further research of Li et al. [13] showed that there is a QTL significantly associated with C10:0 in *ACACA*. In our previous research, a novel SNP in this gene was detected [16], the analysis showed some interaction between the SNP and C13:0, C16:1, and CLA. Pegolo et al. [14] demonstrated that one SNP in *ACACA* was related to some *de novo* synthetized fatty acids: C8:0, C10:0, and C12:0, which were negatively associated with allele A. Interestingly, authors found a slight, but significant relation of this SNP and EPAω3 content. Our results and results from other authors concerning FA saturated forms are consistent with the function of the enzyme encoded by the gene. Unexpectedly, in the presented study, as well as in Pegolo et al. [14] research, the *ACACA* gene was related to some unsaturated, long-chain fatty acids, which are not synthetized in the milk-producing cells. Moreover, we observed no influence of the analyzed SNP on SCFA, which are produced in these cells. These findings are interesting, as the encoded enzyme is responsible for the first step of *de novo* synthesis of fatty acids in the mammary gland.

#### 4.1.2. *SCD1* Gene Effect

The A293V SNP in *SCD1* gene influence on milk traits in cattle is already well described by several authors. Bouwman et al. [11] observed that allele A of this SNP was related with a higher concentration of C10:1, C12:1, and C14:1, and a lower concentration of C10:0, C14:0, and C16:1. Those authors suggest that this SNP effect on medium-chain UFA and their SFA analogues is in accordance with the function of the encoded enzyme. In the presented study, some relations between the SNP versus C10:0 and C12:0 were observed. On the other hand, we found very small effect of the analyzed SNP on FA unsaturation, which is not consistent with the encoded enzyme. Duchemin et al. [10] demonstrated that allele V was negatively associated with C18:0, from C10:1 to C14:1 cis-9 and C18:1 trans-11, C18:3 cis-9,12,15, plus with DI C10 to C14. In opposite, for C8:0 to C14:0, C16:1 cis-9, CLA, and DI C16, a positive relation with this allele was observed. Authors also found a strong effect of A293V on C18:1 trans-11 content. Houaga et al. [18] found significant differences between genotypes for DI C14 and DI total, as well as for C6:0 and C8:0. Their results are very comparable with the results of the presented study. Schennink et al. [6] proposed an explanation of the effect of this SNP, pointing out that this SNP causes substitution of valine to alanine in 293 position which is located in a rich in histidine region of the enzyme, which show strong catalytic activity [19]. As a consequence of this substitution the activity of the encoded enzyme may be altered [6].

#### 4.1.3. *DGAT1* Gene Effect

Another well described SNP affecting FA profile at 50% concentration [6], is K232A mutation in *DGAT1* gene, which is a nonsynonymous non-conservative substitution of lysine to alanine [20]. Bouwman et al. [11] observed that between 63rd and 282nd DIM, allele K was connected with a higher percentage of C6:0, C8:0, C16:0, and C16:1 fractions, as well as with a lower percentage of C14:0, C18:1, and CLA fractions. Our results complete these findings, showing that changes between first phase (<90 DIM) and end of lactation (>210 DIM) in C6:0 and C8:0 content were lower with A allele, while in case of K allele there was clear increase of these FA content (Figure 5). In another study, higher content of C14:1 cis-9, C16:1 cis-9, C18:1 trans-6-8, C18:1 trans-10, C18:2 cis-9 trans-11, plus lower content of C10:0, C16:0 iso, C18:0, C20:0, and C24:0 was observed in KA comparing to AA genotype [7]. Bovenhuis et al. [12] found a highly significantly lower content of C18 FA, such as C18:1 cis-9, CLA cis-9 trans-11, C18:2 cis-9 cis-12, and C18:3 cis-9 cis-12 cis-15 with presence of allele K. Interestingly, our results also indicate a linkage between this SNP and the C18 FA group. We observed that with K allele the content of C18:1 trans-9 was higher, while percentage of FA known as health-oriented components (C18:1 cis-8/11; C18:2 cis-6; C:18ω3) was lower. Moreover, our findings show that K allele is also significantly related to lower PUFA content in milk. It should be noted that our results as well show a linkage between this SNP and the C18 FA group. We observed that with K allele the content of C18:1 trans-9 is higher, while percentage of FA know as health oriented components (C18:1 cis-8/11; C18:2 cis-6; C:18ω3) is lower. Additionally, our findings show that K allele is also significantly related to lower PUFA content in milk.

### 4.2. Changes of FA Content during Lactation Depending on Polymorphism

It is widely known that FA concentration is influenced by many physiological and external factors. Many researches showed concentration changes throughout lactation. Brainbridge et al. [21] demonstrated that lactation stage highly significantly affected FA profile, only two out of 40 analyzed FA remained unaffected (C18:2 cis-9, cis-12; C18:2 cis-9, trans-11). Analyzing FA groups, authors showed that total PUFA, n-6 FA, and total CLA were also unaffected. Bastin et al. [22] demonstrated that UFA content, particularly MUFA, changed during lactation after 100 DIM. Moreover, MCFA and SCFA were more stable than LCFA. Interestingly, authors observed a decrease of C18 FA group (C18:0, C18:1, C18:1 cis, and 18:1 cis-9) during lactation. In the presented research, the FA with 18C atoms (except DI C18) and all FA groups were also affected by lactation phase, but in different ways in different SNPs. Interestingly, the strongest influence of phenotype on FA changes during lactation was detected in case of VV in *SCD1*. The highest increase of unhealthy FA groups and strongest reduction of health-promoting FA groups were observed in presence of this phenotype. Bovenhuis et al. [23] showed significant K232A in *DGAT1* by lactation stage interactions for milk yield, lactose yield, and percentage of fat and protein in milk. It should be noted that in the presented study, this SNP also affected fat and protein percentage changes during lactation, AA phenotype was significantly linked with highest reduction of fat percentage and KK with highest increase of protein percentage during lactation.

## 5. Conclusions

A tendency to increased concentration of unhealthy fatty acids, such as SFA group, and to lower the concentration of healthy fatty acids, such as UFA group, with the presence of homozygous polymorphism: GG in *ACACA*, VV in *SCD1* and KK in *DGAT1* was observed. Interestingly, these phenotypes had the lowest frequency. Moreover, a susceptibility of C18 group to polymorphism of the analyzed genes was observed. Especially interesting is the VV phenotype in *SCD1*, which was related to strongest changes of FA during lactation. The genetic variability and moderate to high heritability of *de novo* synthesized fatty acids give the possibility to modify the FA profile via genetic selection. This with combination of knowledge about non-genetic factors affecting FA such as feeding could lead to the production of functional food. However, displacement of genotypes associated with unhealthy fatty acids, characterized by the lowest frequency, could lead to limit genetic diversity in cattle population as a side effect.

## Figures and Tables

**Table 1:** Allele and genotype frequencies of AJ312201.1g.1488C > G (g.1488C > G) single nucleotide polymorphism (SNP) in acetyl-CoA carboxylase α (*ACACA*) gene, A293V SNP in stearoyl-CoA desaturase (*SCD1*) gene, and K232A SNP in diglyceride acyltransferase (*DGAT1*) gene with χ2 test and *p*-value.

Parameter	*ACACA* g.1488C > G				*SCD1* A293V				*DGAT1* K232A			
Alleles	C		0.78		A		0.77		A		0.72	
Frequencies	G		0.22		V		0.23		K		0.28	
Expected Hardy-Weinberg Frequencies	*n* = 88	CC		0.89	*n* = 82	AA		0.85	*n* = 73	AA		0.74
	*n* = 50	CG		0.49	*n* = 57	VA		0.51	*n* = 61	KA		0.58
	*n* = 6	GG		0.06	*n* = 5	VV		0.08	*n* = 10	KK		0.11
χ2 test	0.11				1.70				0.33			
*p*-value	0.74				0.19				0.57			

*p*-value with 1 degree of freedom.

## Supplementary Materials

The following are available online at https://www.mdpi.com/2076-2615/10/6/997/s1, Table S1. Percentage (mean ± SD) of fatty acids, fat, protein and lactose in milk from Polish Holstein-Frisian cows during 1st and 2nd phase of lactation depending on AJ312201.1g.1488C > G (g.1488C > G) Single Nucleotide Polymorphism (SNP) in Acetyl-CoA Carboxylase α (*ACACA*) gene; Table S2. Percentage (mean ± SD) of fatty acids, fat, protein and lactose in milk from Polish Holstein-Frisian cows during 1st and 2nd phase of lactation depending on A293V Single Nucleotide Polymorphism (SNP) in Stearoyl-CoA Desaturase (*SCD1*) gene. Table S3. Percentage (mean ± SD) of fatty acids, fat, protein and lactose in milk from Polish Holstein-Frisian cows during 1st and 2nd phase of lactation depending on K232A Single Nucleotide Polymorphism (SNP) in Diglyceride Acyltransferase (*DGAT1*) gene.
