# Supplementary material for: Impact of SNPs in ACACA, SCD1, and DGAT1 Genes on Fatty Acid Profile in Bovine Milk with Regard to Lactation Phases
Source: Animals (Basel). 2020 Jun 8;10(6):997. doi: 10.3390/ani10060997 (PMC7341249; doi:10.3390/ani10060997)
Supplement: Supplementary file 1 [file animals-10-00997-s001.pdf]

Table S 1. Percentage (mean ± SD) of fatty acids, fat, protein and lactose in milk from Polish Holstein-Frisian cows during 1st and 2nd phase of lactation depending on AJ312201.1g.1488C>G (g.1488C>G) Single Nucleotide Polymorphism (SNP) in Acetyl-CoA Carboxylase α (*ACACA*) gene.

**Fatty acid (%)**	***ACACA* gene polymorphism**					
	**CC (n = 88)**		**CG (n = 50)**		**GG (n = 6)**	
	**1st**	**2nd**	**1st**	**2nd**	**1st**	**2nd**
C4:0	0.75±0.32	0.74±0.25	0.72±0.30	0.76±0.26	0.67±0.13	0.69±0.17
C6:0	0.89±0.22	0.92±0.20	0.90±0.24	0.93±0.19	0.94±0.16	0.92±0.12
C8:0	0.71^{A}±0.16	0.78^{A}±0.13↑	0.75±0.17	0.78±0.13	0.82±0.17	0.81±0.07
C10:0	1.80^{A}±0.53	2.28^{A}±0.36↑	1.94^{B}±0.53	2.24^{B}±0.40↑	2.30±0.59	2.37±0.32
C12:0	2.12^{A}±0.68	3.15^{A}±0.45↑	2.24^{B}±0.69	3.00^{B}±0.69↑	2.82±0.76	3.28±0.47
C13:0	0.12±0.17	0.11±0.03	0.13±0.17	0.12±0.04	0.16±0.13	0.14±0.10
C14:0	7.71^{A}±1.64	11.04^{A}±0.88↑	8.25^{B}±1.61	10.93^{B}±1.08↑	9.27^{C}±1.26	11.38^{C}±0.93↑
C15:0	0.81^{A}±0.24	1.33^{A}±0.20↑	0.87^{B}±0.19	1.34^{B}±0.27↑	1.07±0.29	1.53±0.52↑
C16:0	27.48^{A}±2.12	30.64^{A}±2.57↑	28.02^{B}±2.19	30.67^{B}±2.99↑	29.26±0.96	29.99±2.60
C17:0	0.69^{A}±0.15	0.57^{A} ±0.09↓	0.67^{B}±0.12	0.58^{B}±0.07↓	0.68±0.10	0.61±0.11
C18:0	11.75^{A}±1.65	8.40^{A}±1.38↓	11.67^{B}±1.67	8.48^{B}±1.60↓	11.75^{a}±1.54	7.67^{a}±1.92↓
C20:0	0.12±0.03	0.12±0.03	0.12±0.04	0.12±0.03	0.15±0.04	0.12±0.04
C14:1	0.79^{A}±0.26	1.59^{A}±0.32↑	0.84^{B}±0.28	1.54^{B}±0.31↑	0.71^{C}±0.31	1.65^{C}±0.36↑
DI C14	0.09^{A}±0.03	0.13^{A}±0.02↑	0.09^{B}±0.02	0.12^{B}±0.02↑	0.07^{C} ±0.03	0.13^{C} ±0.03↑
C16:1	4.46^{A}±1.46	5.67^{A}±1.15↑	4.26^{B}±1.38	5.76^{B}±1.35↑	4.15±1.56	5.59±1.30
DI C16	0.14^{A}±0.05	0.16^{A}±0.03↑	0.13^{B}±0.04	0.16^{B}±0.03↑	0.12±0.04	0.16±0.03
C17:1	0.52^{A}±0.14	0.29^{A}±0.09↓	0.47^{B}±0.14	0.20^{B}±0.09↓	0.43±0.13	0.30±0.12↓
C18:1 *cis*-9	27.81^{A}±4.06	19.21^{A}±2.06↓	29.86^{B}±4.06	19.03^{B}±2.39↓	24.12±5.27	20.58±2.58
C18:1 *cis*-8 (*cis*-11)	1.27^{A}±0.20	0.92^{A}±0.21↓	1.26^{B}±0.19	0.98^{B}±0.25↓	1.11±0.19	1.06±0.26
DI C18	0.70±0.04	0.71±0.04	0.70±0.04	0.70±0.04	0.67±0.04	0.74±0.04

C18:1 *trans*-9	0.84^{A}±0.63	1.08^{A}±0.31↑	0.80^{B}±0.57	1.15^{B}±0.39↑	0.70±0.59	0.92±0.25
C18:1 *trans*-7 (*trans*-11)	1.38^{A}±0.46	1.22^{A}±0.24↓	1.47^{B}±0.60	1.22^{B}±0.25↓	1.21±0.22	1.34±0.22
other *trans* 18:1	0.32±0.13	0.32±0.07	0.30±0.06	0.30±0.07	0.26^{a}±0.05	0.32^{a}±0.06↑
C18:2 *cis*-6	2.44^{A}±0.37	2.11^{A}±0.36↓	2.43^{B}±0.37	2.06^{B}±0.36↓	2.30±0.23	2.19±0.65
CLA	0.41^{A}±0.09	0.50^{A}±0.10↑	0.40^{B}±0.08	0.46^{B}±0.11↑	0.39±0.13	0.48±0.10
C18:3ω3	0.35^{a}±0.06	0.32^{a}±0.09↓	0.34±0.06	0.32±0.08	0.36±0.05	0.31±0.10
C20:1	0.15±0.06	0.15±0.07	0.13±0.06	0.15±0.07	0.12±0.08	0.16±0.07
C20:4ω6	0.15^{A}±0.04	0.18^{A}±0.03↑	0.15^{B}±0.04	0.19^{B}±0.03↑	0.17±0.04	0.19±0.02
SFA	54.95^{A}±4.67	60.07^{A}±3.01↑	56.37^{B}±4.15	59.94^{B}±3.50↑	59.85±3.16	59.50±3.52
MUFA	38.89^{A}±4.27	30.28^{A}±2.28↓	37.71^{B}±4.08	30.26^{B}±2.58↓	34.04±4.21	31.18±2.64
PUFA	3.38^{A}±0.43	3.08^{A}±0.48↓	3.36^{B}±0.40	2.98^{B}±0.45↓	3.24±0.33	3.16±0.81
UFA	42.28^{A}±4.29	33.36^{A}±2.39↓	41.07^{B}±4.07	33.26^{B}±2.68↓	37.28±4.52	34.33±3.31
SCFA	1.64±0.50	1.66±0.45	1.62±0.52	1.69±0.44	1.62±0.28	1.61±0.28
MCFA	46.01^{A}±4.22	56.56^{A}±3.16↑	47.38^{B}±4.36	56.37^{B}±3.92↑	50.52±4.72	56.74±4.50
LCFA	48.23^{A}±4.67	35.37^{A}±3.18↓	47.12^{B}±5.01	35.28^{B}±3.70↓	43.75±6.93	35.64±4.65
trans FA	2.53±0.66	2.63±0.45	2.58±0.74	2.67±0.51	2.17±0.43	2.58±0.27
DI	0.62^{A}±0.10	0.35^{A}±0.03↓	0.59^{B}±0.09	0.35^{B}±0.03↓	0.52^{C}±0.10	0.36^{C}±0.03↓
Fat	4.10^{a}±0.96	3.89^{a}±0.60↓	4.21±1.01	4.10±0.76	3.65±0.35	3.78±0.81
Protein	3.12^{A}±0.26	3.74^{A}±0.32↑	3.14^{B}±0.23	3.74^{B}±0.29↑	3.25^{C}±0.12	3.71^{C}±0.18↑
Lactose	4.96^{A}±0.23	4.76^{A}±0.39↓	4.97^{B}±0.22	4.82^{B}±0.25↓	4.95±0.16	4.86±0.16

DI – Index of Desaturation; SFA – Saturated Fatty Acids; MUFA – Monounsaturated Fatty Acids; UFA – Unsaturated Fatty Acids; SCFA – Short Chain Fatty Acids; MCFA – Medium Chain Fatty Acids; LSFA – Long Chain Fatty Acids; n – number of animals; ↑ - indicates higher content of FA in 2nd phase of lactation comparing to 1st phase of lactation; ↓ - indicates lower content of FA in 2nd phase of lactation comparing to 1st phase of lactation; a, b, c, d – values differ significantly between polymorphisms within rows (P < 0.05); A, B, C, D – values differ highly significantly between polymorphisms within rows (P < 0.01).

Table S 2. Percentage (mean ± SD) of fatty acids, fat, protein and lactose in milk from Polish Holstein-Frisian cows during 1st and 2nd phase of lactation depending on A293V Single Nucleotide Polymorphism (SNP) in Stearoyl-CoA Desaturase (*SCD1*) gene.

**Fatty acid (%)**	***SCD1* gene polymorphism**					
	**AA (n = 82)**		**VA (n = 57)**		**VV (n = 5)**	
	**1st**	**2nd**	**1st**	**2nd**	**1st**	**2nd**
C4:0	0.74±0.33	0.72±0.26	0.72±0.27	0.79±0.22	0.82±0.38	0.63±0.33
C6:0	0.89±0.25	0.90±0.20	0.89^{a}±0.18	0.97^{a}±0.17↑	0.88±0.32	0.87±0.24
C8:0	0.73±0.16	0.76±0.12	0.73^{A}±0.16	0.81^{A}±0.12↑	0.65±0.27	0.76±0.11
C10:0	1.88^{A}±0.48	2.19^{A}±0.35↑	1.89^{B}±0.60	2.38^{B}±0.38↑	1.54±0.72	2.26±0.30
C12:0	2.26^{A}±0.62	3.01^{A}±0.52↑	2.22^{B}±0.79	3.23^{B}±0.50↑	1.78±0.88	3.23±0.46
C13:0	0.12±0.15	0.11±0.03↓	0.13±0.19	0.12±0.04↓	0.08±0.08	0.16±0.09
C14:0	8.10^{A}±1.48	11.92^{A}±0.86↑	7.65^{B}±1.82	11.15^{B}±1.08↑	6.93^{a}±1.92	11.06^{a}±0.54↑
C15:0	0.87^{A}±0.22	1.35^{A}±0.19↑	0.80^{B}±0.25	1.32^{B}±0.28↑	0.75^{a}±0.26	1.50^{a}±0.59↑
C16:0	27.79^{A}±2.14	30.80^{A}±2.71↑	27.73^{B}±2.15	30.31^{B}±2.74↑	26.90^{a}±2.05	31.46^{a}±2.48↑
C17:0	0.69^{A}±0.15	0.57^{A}±0.08↓	0.67^{B}±0.12	0.58^{B}±0.07↓	0.80^{a}±0.19	0.56^{a}±0.11↓
C18:0	11.52^{A}±1.55	8.28^{A}±1.38↓	11.89^{B}±1.69	8.64^{B}±1.60↓	13.24^{C}±1.90	7.49^{C}±1.21↓
C20:0	0.12±0.04	0.12±0.03	0.12±0.03	0.12±0.03	0.13±0.05	0.10±0.02
C14:1	0.87^{A}±0.27	1.67^{A}±0.34↑	0.71^{B}±0.24	1.46^{B}±0.24↑	0.69^{a}±0.28	1.47^{a}±0.27↑
DI C14	0.10^{A}±0.02	0.13^{A}±0.02↑	0.08^{B}±0.02	0.12^{B}±0.02↑	0.09^{a}±0.02	0.12^{a}±0.02↑
C16:1	4.27^{A}±1.42	5.63^{A}±1.20↑	4.55^{B}±1.46	5.65^{B}±1.25↑	4.24^{C}±1.39	6.75^{C}±0.92↑
DI C16	0.13^{A}±0.04	0.15^{A}±0.03↑	0.14^{a}±0.04	0.16^{a}±0.03↑	0.14^{b}±0.05	0.18^{b}±0.02↑
C17:1	0.49^{A}±0.13	0.28^{A}±0.09↓	0.49^{B}±0.16	0.30^{B}±0.09↓	0.57^{a}±0.09	0.32^{a}±0.07↓
C18:1 *cis*-9	27.26^{A}±3.92	19.38^{A}±2.25↓	27.28^{B}±4.59	19.02^{B}±2.10↓	26.87^{C}±2.97	17.67^{C}±1.71↓
C18:1 *cis*-8 (*cis*-11)	1.27^{A}±0.19	0.96^{A}±0.23↓	1.26^{B}±0.22	0.94^{B}±0.23↓	1.22^{a}±0.11	0.87^{a}±0.22↓
DI C18	0.70±0.04	0.71±0.04	0.69±0.04	0.70±0.04	0.69±0.03	0.71±0.02
C18:1 *trans*-9	0.80^{A}±0.62	1.07^{A}±0.32↑	0.86^{B}±0.58	1.13^{B}±0.36↑	0.70±0.70	1.26±0.29

C18:1 *trans*-7 (*trans*-11)	1.48^{A}±0.59	1.23^{A}±0.24↓	1.28±0.36	1.22±0.25↓	1.59^{B}±0.14	1.14^{B}±0.15↓
other *trans* 18:1	0.30^{a}±0.08	0.33^{a}±0.08↑	0.30±0.09	0.30±0.06	0.52±0.41	0.28±0.07
C18:2 *cis*-6	2.48^{A}±0.38	2.15^{A}±0.39↓	2.36^{B}±0.36	2.05^{B}±0.34↓	2.41^{a}±0.24	1.78^{a}±0.16↓
CLA	0.41^{A}±0.09	0.48^{A}±0.11↑	2.36^{B}±036	2.06^{B}±0.34↓	0.42±0.07	0.46±0.06
C18:3ω3	0.35±0.06	0.33±0.10	0.34^{a}±0.06	0.31^{a}±0.06↓	0.36^{b}±0.09	0.27^{b}±0.05↓
C20:1	0.15±0.06	0.15±0.07	0.14±0.06	0.16±0.07	0.11±0.09	0.14±0.07
C20:4ω6	0.15^{A}±0.04	0.19^{A}±0.03↑	0.15^{B}±0.04	0.18^{B}±0.03↑	0.12±0.05	0.17±0.05
SFA	55.73^{A}±4.38	59.71^{A}±3.21↑	55.63^{B}±4.84	60.41^{B}±3.21↑	54.49±4.72	60.07±2.24
MUFA	38.24^{A}±4.09	30.55^{A}±2.46↓	38.21^{B}±4.69	30.02^{B}±2.34↓	39.75^{C}±3.43	29.71^{C}±1.44↓
PUFA	3.43^{A}±0.42	3.12^{A}±0.52↓	3.28^{B}±0.40	2.99^{B}±0.42↓	3.37^{C}±0.32	2.63^{C}±0.18↓
UFA	41.67^{A}±4.15	33.67^{A}±2.63↓	41.49^{B}±4.70	33.01^{B}±2.40↓	43.12^{a}±3.37	32.34^{a}±1.32↓
SCFA	1.64±0.55	1.62±0.46	1.61±0.39	1.76±0.38	1.69±0.68	1.49±0.57
MCFA	46.91^{A}±4.11	56.43^{A}±3.33↑	46.60^{B}±4.68	56.42^{B}±3.71↑	43.56^{C}±4.88	58.66^{C}±2.73↑
LCFA	47.51^{A}±4.56	35.49^{A}±3.30↓	47.57^{B}±5.49	35.41^{B}±3.53↓	51.02^{C}±4.24	32.41^{C}±3.18↓
trans FA	2.58±0.73	2.64±0.45	2.44^{a}±0.60	2.65^{a}±0.50↑	2.67±0.70	2.62±0.38
DI	0.61^{A}±0.10	0.35^{A}±0.03↓	0.61^{B}±0.11	0.34^{B}±0.03↓	0.64^{C}±0.09	0.34^{C}±0.01↓
Fat	4.01±1.03	3.93±0.72	4.30^{a}±0.88	4.02^{a}±0.62↓	3.95±0.74	3.83±0.37
Protein	3.11^{A}±0.26	3.72^{A}±0.32↑	3.15^{B}±0.24	3.76 ±0.29↑	3.13^{C}±0.17	3.71^{C}±0.16↑
Lactose	4.96^{A}±0.24	4.81^{A}±0.30↓	4.97^{B}±0.20	4.75^{B}±0.39↓	4.99±0.13	4.70±0.26

DI – Index of Desaturation; SFA – Saturated Fatty Acids; MUFA – Monounsaturated Fatty Acids; UFA – Unsaturated Fatty Acids; SCFA – Short Chain Fatty Acids; MCFA – Medium Chain Fatty Acids; LSFA – Long Chain Fatty Acids; n – number of animals; ↑ - indicates higher content of FA in 2nd phase of lactation comparing to 1st phase of lactation; ↓ - indicates lower content of FA in 2nd phase of lactation comparing to 1st phase of lactation; a, b, c, d – values differ significantly between polymorphisms within rows ($P < 0.05$); A, B, C, D – values differ highly significantly between polymorphisms within rows ($P < 0.01$).

Table S 3. Percentage (mean ± SD) of fatty acids, fat, protein and lactose in milk from Polish Holstein-Frisian cows during 1st and 2nd phase of lactation depending on K232A Single Nucleotide Polymorphism (SNP) in Diglyceride Acyltransferase (*DGAT1*) gene.

**Fatty acid (%)**	***DGAT1* gene polymorphism**					
	**AA (n = 73)**		**KA (n = 61)**		**KK (n = 10)**	
	**1st**	**2nd**	**1st**	**2nd**	**1st**	**2nd**
C4:0	0.74±0.32	0.73±0.24	0.76±0.27	0.74±0.26	0.56^{a}±0.33	0.86^{a}±0.23↑
C6:0	0.86±0.23	0.90±0.18	0.94±0.23	0.93±0.20	0.80^{a}±0.15	1.06^{a}±0.18↑
C8:0	0.70^{A}±0.16	0.77^{A}±0.11↑	0.76±0.17	0.79±0.13	0.73^{a}±0.11	0.88^{a}±0.12↑
C10:0	1.81^{A}±0.52	2.25^{A}±0.36↑	1.92^{B}±0.56	2.26^{B}±0.39↑	1.98^{a}±0.48	2.46^{a}±0.35↑
C12:0	2.15^{A}±0.67	3.07^{A}±0.61↑	2.29^{B}±0.74	3.10^{B}±0.49↑	2.41^{C}±0.65	3.35^{C}±0.38↑
C13:0	0.13±0.17	0.12±0.05	0.12±0.17	0.11±0.03	0.08±0.06	0.11±0.03
C14:0	7.79^{A}±1.51	11.20^{A}±0.99↑	8.14^{B}±1.84	10.85^{B}±0.91↑	8.16^{C}±1.31	10.60^{C}±0.64↑
C15:0	0.84^{A}±0.22	1.37^{A}±0.29↑	0.83^{B}±0.21	1.32^{B}±0.19↑	0.89^{a}±0.38	1.24^{a}±0.21↑
C16:0	27.22^{A}±1.85	30.22^{A}±2.79↑	28.29^{B}±2.27	31.10^{B}±2.59↑	28.15±2.54	30.66±2.66↑
C17:0	0.69^{A}±0.12	0.59^{A}±0.07↓	0.67^{B}±0.12	0.55^{B}±0.09↓	0.73±0.33	0.58±0.06
C18:0	11.88^{A}±1.71	8.54^{A}±1.63↓	11.70^{B}±1.57	8.23^{B}±1.30↓	10.70^{C}±1.32	8.31^{C}±1.35↓
C20:0	0.13±0.03	0.12±0.03	0.12±0.04	0.12±0.02	0.12±0.04	0.12±0.03
C14:1	0.76^{A}±0.27	1.57^{A}±0.36↑	0.87^{B}±0.26	1.60^{B}±0.28↑	0.73^{C}±0.19	1.49^{C}±0.18↑
DI C14	0.09^{A}±0.03	0.12^{A}±0.02↑	0.10^{B}±0.02	0.13^{B}±0.02↑	0.08^{C}±0.02	0.12^{C}±0.01↑
C16:1	4.59^{A}±1.44	5.47^{A}±1.22↑	3.99^{B}±1.28	5.88^{B}±1.22↑	5.26±1.68	5.90±1.13
DI C16	0.14±0.04	0.15±0.03	0.12^{A}±0.04	0.16^{A}±0.03↑	0.16±0.05	0.16±0.03
C17:1	0.50^{A}±0.14	0.29^{A}±0.09↓	0.50^{B}±0.15	0.30^{B}±0.09↓	0.45^{C}±0.12	0.27^{C}±0.08↓
C18:1 *cis*-9	22.38^{A}±4.05	19.36^{A}±2.19↓	22.43^{B}±4.18	19.05^{B}±2.25↓	26.28^{C}±5.07	18.66^{C}±1.94↓
C18:1 *cis*-8 (*cis*-11)	1.27^{A}±0.20	0.99^{A}±0.25↓	1.27^{B}±0.20	0.92^{B}±0.20↓	1.13^{C}±0.17	0.78^{C}±0.14↓
DI C18	0.70±0.04	0.71±0.04	0.70±0.04	0.71±0.04	0.71±0.04	0.70±0.03
C18:1 *trans*-9	0.92^{a}±0.63	1.09^{a}±0.36↑	0.67^{A}±0.52	1.11^{A}±0.32↑	1.08±0.72	1.14±0.35

C18:1 *trans*-7 (*trans*-11)	1.45^{a}±0.62	1.27^{a}±0.25↓	1.40^{A}±0.35	1.10^{A}±0.23↓	1.10±0.23	1.17±0.16
other *trans* 18:1	0.31±0.13	0.31±0.07	0.31±0.09	0.33±0.07	0.28±0.08	0.29±0.05
C18:2 *cis*-6	2.42^{A}±0.38	2.18^{A}±0.37↓	2.49^{B}±0.34	2.01^{B}±0.37↓	2.12±0.27	2.00±0.31
CLA	0.41^{A}±0.10	0.48^{A}±0.10↑	0.42^{B}±0.08	0.48^{B}±0.12↑	0.35^{C}±0.05	0.48^{C}±0.09↑
C18:3ω3	0.35±0.06	0.35±0.09	0.34^{A}±0.06	0.30^{A}±0.07↓	0.31±0.07	0.28±0.07
C20:1	0.14±0.06	0.14±0.07	0.16±0.06	0.16±0.06	0.12±0.06	0.15±0.06
C20:4ω6	0.15^{A}±0.04	0.19^{A}±0.03↑	0.15^{B}±0.04	0.18^{B}±0.03↑	0.16±0.04	0.18±0.02
SFA	54.95^{A}±4.28	59.88^{A}±3.09↑	56.54^{B}±4.82	60.11^{B}±3.35↑	55.32^{a}±4.27	60.21^{a}±3.12↑
MUFA	38.63^{A}±4.16	30.34^{A}±2.31↓	37.95^{B}±4.48	30.38^{B}±2.57↓	37.75^{C}±4.44	29.69^{C}±2.20↓
PUFA	3.37^{A}±0.42	3.16^{A}±0.49↓	3.48^{B}±0.39	2.94^{B}±0.47↓	2.97±0.33	2.93±0.42↓
UFA	42.00^{A}±4.26	33.50^{A}±2.47↓	41.38^{B}±4.43	33.32^{B}±2.61↓	40.71^{C}±4.40	32.62^{C}±2.43↓
SCFA	1.60±0.52	1.63±0.42	1.71±0.47	1.67±0.46	1.36^{a}±0.44	1.91^{a}±0.41↑
MCFA	45.99^{A}±3.85	56.04^{A}±3.50↑	47.20^{B}±4.85	57.03^{B}±3.37↑	48.39^{C}±4.49	56.68^{C}±3.76↑
LCFA	48.01^{A}±4.64	35.86^{A}±3.49↓	47.67^{B}±5.17	34.00^{B}±3.25↓	44.95^{C}±5.43	34.39^{C}±3.51↓
trans FA	2.66±0.76	2.67±0.52	2.39^{A}±0.55	2.62^{A}±0.41↑	2.45±0.66	2.59±0.39
DI	0.62^{A}±0.10	0.35^{A}±0.03↓	0.60^{B}±0.10	0.35^{B}±0.03↓	0.60^{C}±0.11	0.34^{C}±0.02↓
Fat	4.03^{a}±1.00	3.77^{a}±0.62↓	4.15±0.92	4.14±0.63	4.63±0.92	4.26±0.90
Protein	3.11^{A}±0.27	3.67^{A}±0.30↑	3.14^{B}±0.22	3.78^{B}±0.27↑	3.20^{C}±0.22	3.96^{C}±0.39↑
Lactose	4.96^{A}±0.21	4.78^{A}±0.34↓	4.98^{B}±0.24	4.80^{B}±0.32↓	4.91±0.24	4.74±0.45

DI – Index of Desaturation; SFA – Saturated Fatty Acids; MUFA – Monounsaturated Fatty Acids; UFA – Unsaturated Fatty Acids; SCFA – Short Chain Fatty Acids; MCFA – Medium Chain Fatty Acids; LSFA – Long Chain Fatty Acids; n – number of animals; ↑ - indicates higher content of FA in 2nd phase of lactation comparing to 1st phase of lactation; ↓ - indicates lower content of FA in 2nd phase of lactation comparing to 1st phase of lactation; a, b, c, d – values differ significantly between polymorphisms within rows ($P < 0.05$); A, B, C, D – values differ highly significantly between polymorphisms within rows ($P < 0.01$).
